# Clinical presentation, maternal-fetal, and neonatal outcomes of early-onset versus late onset preeclampsia-eclampsia syndrome in a teaching hospital in a low-resource setting: A retrospective cohort study

**DOI:** 10.1371/journal.pone.0281952

**Published:** 2023-02-27

**Authors:** Hale Teka, Awol Yemane, Hiluf Ebuy Abraha, Ephrem Berhe, Habtom Tadesse, Fanos Gebru, Mohammedtahir Yahya, Ytbarek Tadesse, Daniel Gebre, Marta Abrha, Bisrat Tesfay, Ashenafi Tekle, Tsega Gebremariam, Birhane Amare, Mohamedawel Mohamedniguss Ebrahim, Yibrah Berhe Zelelow, Afework Mulugeta

**Affiliations:** 1 Department of Obstetrics and Gynecology, School of Medicine, Mekelle University, Mek’ele, Ethiopia; 2 Ayder Comprehensive Specialized Hospital, Quality Assurance Office, Mekelle University, Mek’ele, Ethiopia; 3 Department of Internal Medicine, School of Medicine, Mekelle University, Mek’ele, Ethiopia; 4 Department of Midwifery, Ayder Comprehensive Specialised Hospital, Mekelle University, Mek’ele, Ethiopia; 5 Department of Nutrition, School of Public Health, Mekelle University, Mek’ele, Ethiopia; University of Gondar, ETHIOPIA

## Abstract

**Background:**

Pre-eclampsia-eclampsia syndrome remains the leading cause of maternal and neonatal mortality worldwide. Both from pathophysiologic and clinical stand points, early and late onset preeclampsia are thought to be two different disease entities. However, the magnitude of preeclampsia-eclampsia and maternal-fetal and neonatal outcomes of early and late onset preeclampsia are not adequately investigated in resource-limited settings. This study sought to examine the clinical presentation and maternal-fetal and neonatal outcome of these two entities of the disease in Ayder comprehensive specialized hospital, an academic setting in Tigray, Ethiopia, from January 1, 2015—December 31, 2021.

**Methods:**

A retrospective cohort design was employed. The patient charts were reviewed to see the baseline characteristics and their progress from the onset of the disease in the antepartum, intrapartum and postpartum periods. Women who developed pre-eclampsia before 34 weeks of gestation were defined as having early-onset pre-eclampsia, and those who developed at 34 weeks or later were identified as late-onset preeclampsia. We used chi-square, t-test and multivariable logistic regression analyses to determine differences between early- and late onset diseases in terms of clinical presentation, maternal-fetal, and neonatal outcomes.

**Results:**

Among the 27,350 mothers who gave birth at the Ayder comprehensive specialized hospital, 1095 mothers had preeclampsia-eclampsia syndrome, with a prevalence of 4.0% (95% CI: 3.8, 4.2)]. Of the 934 mothers analyzed early and late onset diseases accounted for 253 (27.1%) and 681 (72.9%) respectively. Overall, death of 25 mothers was recorded. Women with early onset disease had significant unfavorable maternal outcomes including having preeclampsia with severity features (AOR = 2.92, 95% CI: 1.92, 4.45), liver dysfunction (AOR = 1.75, 95% CI: 1.04, 2.95), uncontrolled diastolic blood pressure (AOR = 1.71, 95% CI: 1.03, 2.84), and prolonged hospitalization (AOR = 4.70, 95% CI: 2.15, 10.28). Similarly, they also had increased unfavorable perinatal outcomes, including the APGAR score at the 5^th^ minute (AOR = 13.79, 95% CI: 1.16, 163.78), low birth weight (AOR = 10.14, 95% CI 4.29, 23.91), and neonatal death (AOR = 6.82, 95% CI: 1.89, 24.58).

**Conclusion:**

The present study highlights the clinical differences between early versus late onset preeclampsia. Women with early-onset disease are at increased levels of unfavorable maternal outcomes. Perinatal morbidity and mortality were also increased significantly in women with early onset disease. Therefore, gestational age at the onset of the disease should be taken as an important indicator of the severity of the disease with unfavorable maternal, fetal, and neonatal outcomes.

## Introduction

Hypertensive disorders of pregnancy complicate 5–10% of pregnancies and remain the most important cause of maternal and perinatal mortality [1, 2]. According to the International Society for the Study of Hypertension in Pregnancy (ISSHP), hypertensive disorders of pregnancy are classified as “chronic (predating pregnancy or diagnosed before 20 weeks of pregnancy) or de novo (either preeclampsia/eclampsia or gestational hypertension)” [3]. According to this classification, preeclampsia-eclampsia are considered as a syndrome, and preeclampsia can be de novo or superimposed on chronic hypertension. Pre-eclampsia-eclampsia syndrome (PE-E) is a multi-system disease that contributes to an annual average of roughly 60,000–80,000 maternal deaths globally [4, 5]. Severe Preeclampsia and eclampsia have considerable adverse impacts on maternal, fetal, and neonatal health, especially in low-resource countries [6, 7]. PE-E syndrome form a deadly trio with obstetric hemorrhage and sepsis resulting in avoidable maternal and neonatal mortality in low-resource settings [8].

Several factors affect maternal, fetal, and neonatal morbidity and mortality associated with PE syndrome; one among these factors is the time of the onset of the disease, ie, early Vs late onset [9]. Early onset preeclampsia (EO-PE) is when preeclampsia occurs at <34 weeks gestation, and late onset preeclampsia (LO-PE) is when preeclampsia occurs at ≥ 34 weeks gestation [10]. Early and late onset preeclampsia are thought to be two different disease entities in terms of pathophysiology and clinical outcome [11]. Early detection and management of both types of preeclampsia is essential. Delayed detection and treatment of pre-eclampsia negatively affect both maternal and neonatal outcomes [12].

However, research studies comparing the adverse maternal, feta land neonatal outcomes of early and late onset PE-E are limited and therefore little is known on the differences in maternal-fetal complications of EO-PE and LO-PE in low-resource settings. Thus, the retrospective study was designed to find out the prevalence of early and late-onset PE-E and to compare the maternal, feta land neonatal outcomes of early and late onset PE-E among women who visited Ayder Comprehensive Specialized Hospital (ACHS) in Tigray, Ethiopia, from January 1, 2015 to December 31, 2021.

## Methods

### Study design

A retrospective cohort design was used to examine clinical presentation, maternal outcome, and neonatal outcomes among women with EO-PE and LO-PE. It is a review of records of antenatal visits, inpatient, intrapartum, and postpartum visits of mothers with preeclampsia-eclampsia over a 7-year period from January 1, 2015 –December 31, 2021.

### Study setting

This study was carried out at the Ayder comprehensive specialized hospital, a teaching hospital in the Tigray region of Ethiopia. It is the largest referral center in the Tigray region for a population of more than 8 million people from Tigray and neighboring districts of the Afar and Amhara regions. The hospital provides comprehensive specialty obstetrics and gynecology services that are among the main services offered at the center. It houses two separate antenatal care clinics for low- and high-risk patients. The number of deliveries has been increasing from time, to time and currently the hospital hosts an average of 5000 deliveries per annum. It also has a neonatal care unit that receives referral both from the maternity ward and other institutions.

### Study population

The study population were records of all women who received antenatal care, intrapartum care, and postnatal care at Ayder hospital or who were referred to Ayder comprehensive specialized hospital during or following delivery, and who had pre-eclampsia-eclampsia syndrome during the study period (January 1, 2015 to December 31, 2021).

### Eligibility criteria

Preeclamptic women who visited Ayder comprehensive specialized hospital between January 1, 2015 and December 31, 2021 were included in this study. Women who had preeclampsia-eclampsia syndrome and their pregnancy terminated before the age of viability, i.e., gestational age <28 weeks and charts with incomplete records were excluded.

### Outcomes and conceptual framework

**Fig 1:**
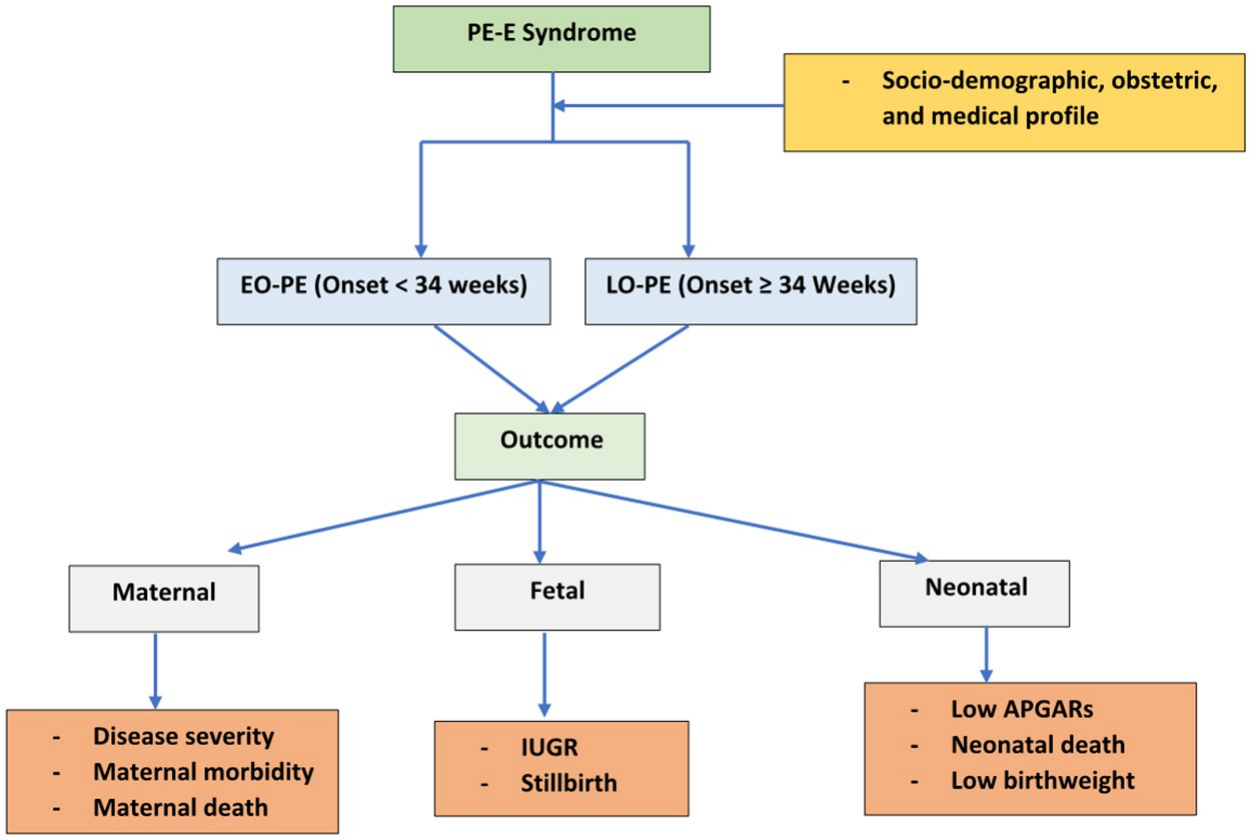
Conceptual framework to guide the retrospective review of records of women who experienced EO-PE or LO-PE. PE-E: Preeclampsia-eclampsia, EO-PE: Early onset preeclampsia, LO-PE: Late-onset Preeclampsia, IUGR: Intrauterine growth restriction.

The study attempted to compare the association of EO-PE vs LO-PE with adverse maternal, fetal, and neonatal outcome. We compared the prevalence of severe disease, maternal morbidity and mortality, fetal and neonatal morbidity and mortality between EO-PE and LO-PE. The association between disease severity, maternal and neonatal complications and EO-PE and LO-PE was estimated and compared. We presented a conceptual framework to guide the retrospective review of records of women who experienced EO-PE or LO- PE (Fig 1).

### Sample-size and sampling techniques

**Fig 2:**
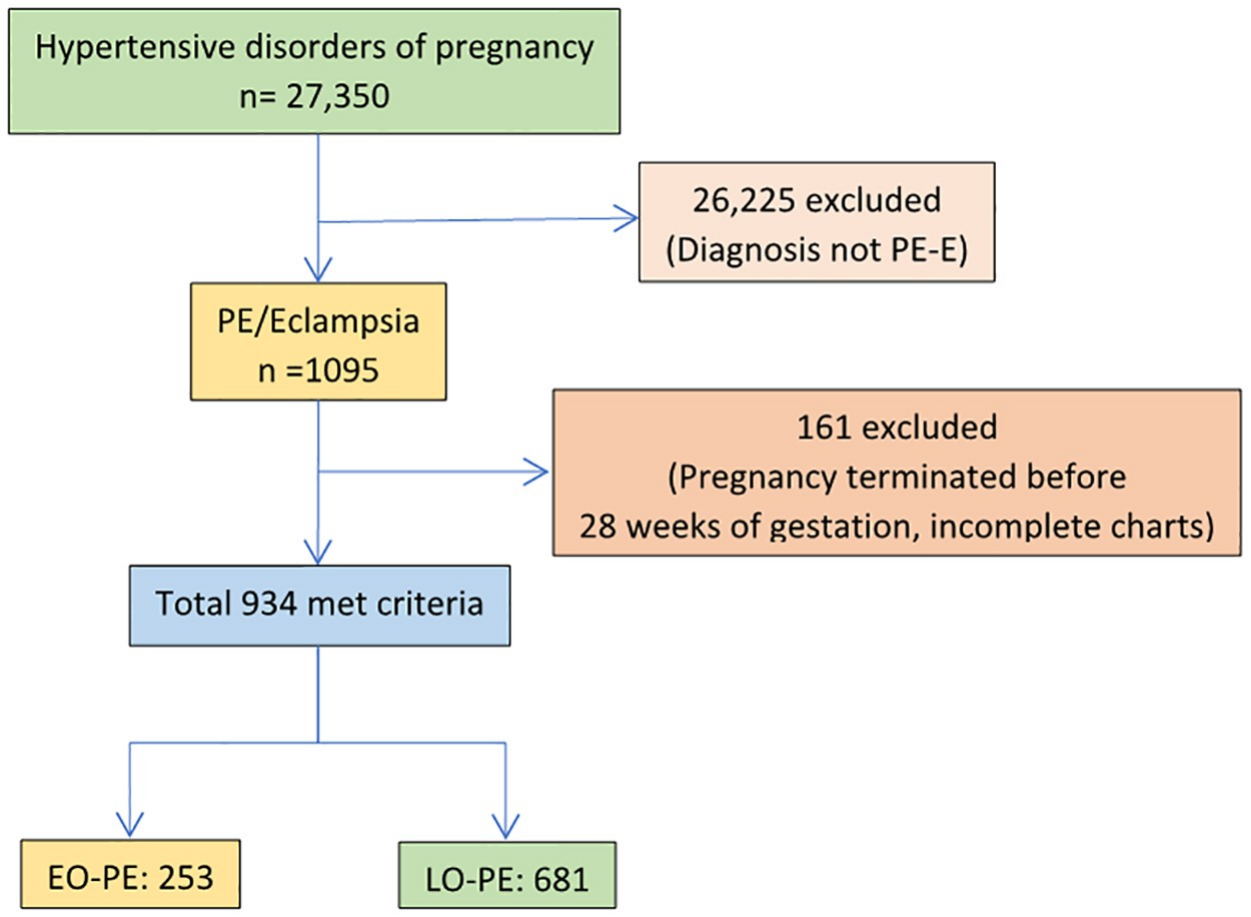
Flow diagram of study participants, Ayder comprehensive specialized hospital, Mekelle, Northern Ethiopia, 2015–2021.

An a priori power analysis was performed to determine an adequate sample size for our primary outcome, maternal complications. We estimate that a minimum sample size of 620 women included in the study by Ndwiga et al. [12] would provide greater than 80% of the ability to identify the difference between early and late gestational age at presentation in the primary outcome of maternal hospitalization after seven days (OR = 5.8; CI 3.9–8.4; p<0.001). In this study, we included 934 consecutively collected preeclamptic women who fulfilled the inclusion criteria (Fig 2).

### Operational definitions

According to the ISSHP 2021 classification, preeclampsia is defined as gestational hypertension accompanied by one or more of the following new- onset conditions at ≥20 weeks’ gestation: 1) proteinuria 2) maternal end organ dysfunction and 3) placental dysfunction [3]. Eclampsia is defined as the occurrence of generalized seizures and/or loss of consciousness generally in addition to preeclampsia criteria [13].

Early onset Preeclampsia (EO-PE) is when preeclampsia occurs at <34 weeks gestation, and late onset preeclampsia (LO-PE) is when preeclampsia occurs at ≥ 34 weeks gestation [9].

Preeclampsia with severity features was diagnosed when one or more of the following maternal systemic complications accompanied hypertension: platelet count < 100,000, mmol/L, aspartate transaminase (AST) > 62, alanine transaminase (ALT) > 64, headache, right upper quadrant pain, epigastric pain, vomiting, hand swelling, facial swelling, abdominal swelling, vulvar edema, blurred vision, blindness, or systolic BP ≥ 160 and/ or diastolic BP ≥ 110 mmHg.

Maternal complications include one or more of the following manifestations: renal insufficiency (creatinine > 1.2), liver involvement (aspartate transaminase (AST) > 62 or alanine transaminase (ALT) > 64), neurologic complication (abnormal body movement, headache, or blurred vision), hematologic complication (Hgb < 10 g/dl and hemolysis). For the liver function test derangement, we took twice the above normal of our institution’s laboratory value. In hospitals’ laboratories the upper normal for AST and ALT is 31 and 32 respectively.

Employing the ICD-10 version 2016, maternal death was defined as “A maternal death is the death of a woman while pregnant or within 42 days of termination of pregnancy, irrespective of the duration and the site of the pregnancy, from any cause related to or aggravated by the pregnancy or its management, but not from accidental or incidental causes” [14].

Pregnancy outcome includes intrauterine growth restriction (IUGR) and stillbirth. The neonatal complication includes one or more of the following conditions: 5-minute APGAR less than 7, birth weight less than 2.5 kg, or neonatal death. In the study area, the period of viability is defined at a gestational age of 28 weeks or above [15].

IUGR is defined as an estimated fetal weight less than the 10th percentile for gestational age by prenatal ultrasound evaluation with additional Doppler abnormalities in utero or physical abnormalities observed afterbirth [16].

Neonatal death is defined as ‘the death of a live born infant, regardless of gestational age at birth, within the first 28 completed days of life” [17].

### Clinical and laboratory data

The management and follow-up protocol for the management of preeclampsia and eclampsia syndrome of patients within the reviewed charts is presented here. All patients were evaluated and the clinical and laboratory findings recorded in their charts by Obstetrics and Gynecology resident physicians. The senior Obstetricians and Gynecologists were involved in the management decision of all patients. The following clinical and laboratory data at initial presentation were recorded on the charts: address, age, parity, gestational age, presence/absence of severity features, blood pressure, AST, ALT, creatinine, and complete blood count. The following symptoms of preeclampsia were also recorded during each follow-up: headache, right upper quadrant pain, epigastric pain, vomiting, hand swelling, facial swelling, abdominal swelling, vulvar edema, blurred vision, blindness, and nasal bleeding. The following laboratory data during follow up were additionally recorded: creatinine, AST, ALT, and complete blood count. Women with preeclampsia with severity features were admitted to maternity ward and reviewed for symptoms on daily basis while laboratory was reviewed weekly. The patients were kept for 48 hours after delivery for follow-up unless they develop complications.

Labor follow-up in the study setting was with intermittent auscultation. Decisions were made for cesarean section for fetal indication for baseline fetal tachycardia and fetal bradycardia. The local protocol for the management of preeclampsia–eclampsia syndrome and the preeclampsia chart are attached as a supplement.

### Data analysis

Data were entered into the open data kit tool; it was analyzed using Stata version 16 statistical software. Descriptive statistics were reported using frequency and percentage. The central tendency and dispersion were estimated using the mean with its standard deviation (SD) or median with its interquartile range (IQR), depending on its normality status. For categorical variables, comparisons were made using the Chi-square test or Fisher’s exact test, as appropriate. Continuous variables were compared using an independent t-test.

Bivariate logistic regression analysis was conducted to see the association between the onset of preeclampsia and the independent variables. Variables that showed an association with the onset of preeclampsia (with a p value <0.25) were included in multivariable logistic regression to see if there was a significant association between each independent variable and the time of preeclampsia onset. Finally, independent variables associated with the onset of preeclampsia at P-value<0.05 were considered statistically significant. Multicollinearity diagnostics was performed and collinear variables were excluded from the final model. Fitness of the final model was checked using the Hosmer-Lemeshow goodness of fit model.

### Ethical clearance

Ethical approval was obtained from the Institutional Review Board (IRB) of Mekelle University, College of Health Sciences and the ethical approval number is: MU-IRB 2016/2022. Since this was a retrospective study, consent from patients could not be obtained. However, the patient’s profile and patient data were fully anonymized. After reviewing the protocol for this study, the IRB waived the requirement of informed consent.

## Results

### Demographic and clinical characteristics

In the seven-year period, a total of 27,350 babies were delivered in the Ayder comprehensive specialized hospital, among them 1095 had preeclampsia-eclampsia syndrome. Of these, 161 (14.7%) of them were excluded because they did not fulfill the eligibility criteria. The prevalence of preeclampsia was [4.0 (95% CI: 3.8, 4.2)]. Finally, 934 (253 with early and 681 late-onset preeclampsia) participants were included in the analysis.

Similar baseline characteristics were observed among women with early- and late-onset preeclampsia. The mean age of the participants was 27.4 (SD, 6.3). More than half (61.6%) of the women were urban inhabitants. A total of 404 (43.2%) mothers were gravida one (range, 0–12) and para one (range, 1–12). A total of 93.2% of women had antenatal care follow-up (Table 1).

**Table 1:** Demographic and clinical characteristics of study participants, Ayder comprehensive special hospital, Mekelle, Northern Ethiopia, 2017–2021 (n = 934).

Characteristic	Total (n = 934)	Onset		P-value
		Early (n = 253)	Late (n = 681)	
Age in years [mean (SD)]	27.4 (6.3)	28.0 (6.7)	27.2 (6.2)	0.08
Residence, n (%)				
Urban	575 (61.6)	150 (59.3)	425 (62.4)	0.384
Rural	359 (38.4)	103 (40.7)	256 (37.6)	
Parity, n (%) (range, 0–12)				
0	404 (43.2)	105 (41.5)	299 (43.9)	0.634
1	40 (4.3)	10 (4.0)	30 (4.4)	
2	167 (17.9)	40 (15.8)	127 (18.6)	
3+	323 (34.6)	98 (38.7)	225 (33.1)	
Gravidity, n (%) (range, 1–12)				
1	404 (43.2)	105 (41.5)	299 (43.9)	0.588
2	184 (19.7)	45 (17.8)	139 (20.4)	
3	112 (12.0)	33 (13.0)	79 (11.6)	
4+	234 (25.1)	70 (27.7)	164 (24.1)	
Admission SBP [mean (SD)]	150.3 (21.0)	150.2 (23.4)	150.3 (20.1)	0.963
Admission DBP [mean (SD)]	103.4 (72.1)	104.7 (76.6)	102.8 (70.4)	0.730
ANC follow-up, n (%)				
Yes	870 (93.2)	233 (92.1)	637 (93.5)	0.603
No	64 (6.8)	20 (7.9)	44 (6.5)	

SD: standard deviation, GA: gestational age, SBP: systolic blood pressure, DBP: diastolic blood pressure.

### Risk factors for the onset of preeclampsia

**Fig 3:**
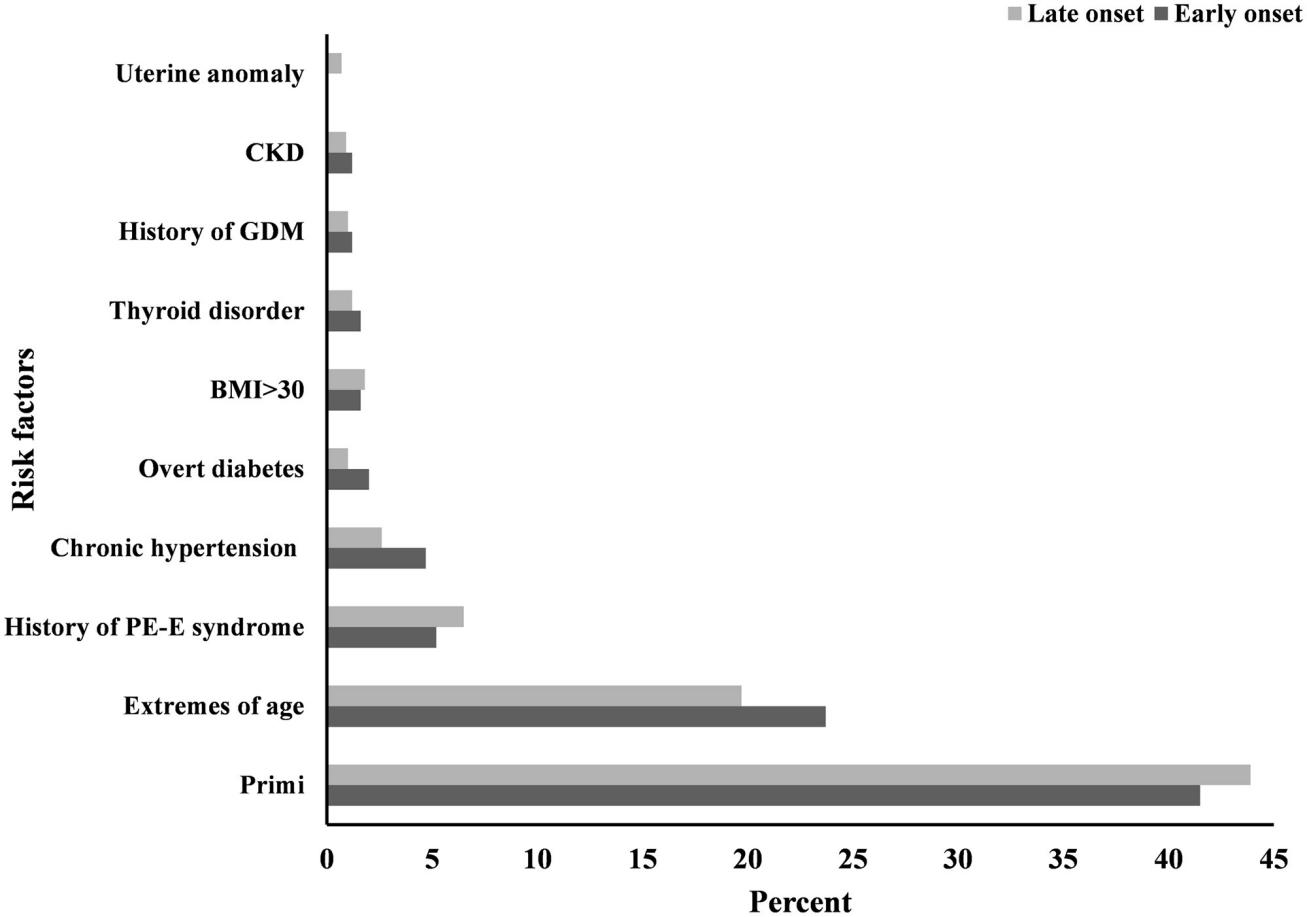
Risk factors for the onset of preeclampsia, Ayder comprehensive specimen, Mekelle, Northern Ethiopia, 2017–2021 (n = 934). CKD: chronic kidney disease, BMI: body mass index, PE-E: preeclampsia-eclampsia.

Mothers in the early onset preeclampsia group were more in the extreme age group (< = 18 or > = 35) as compared to mothers on late onset preeclampsia. In total, 57 (6.1%) women had a history of preeclampsia-eclampsia syndrome. Superimposed preeclampsia was observed in 30 (3.2%) of the participants. Twelve (1.3%) of the participants had overt diabetes; and a body mass index (BMI) greater than 30 was measured in 16 (1.7%) of the women. However, none of the risk factors showed statistically significant association with either of the two groups (Fig 3).

### Severity features of preeclampsia

Preeclampsia with severity features was more commonly seen among women with early onset compared to those with late onset preeclampsia (AOR = 2.92, 95% CI 1.92, 4.45). Of the severity features, headache (AOR = 1.52, 95% CI: 1.01, 2.28) and facial swelling (AOR = 2.73, 95% CI: 1.17, 6.39) were more experienced by mothers with early-onset preeclampsia. Higher diastolic blood pressure was also observed among mothers with early-onset preeclampsia (AOR = 1.71, 95% CI 1.03, 2.84). A higher aspartate aminotransferase (AST) value was observed more among women with early-onset preeclampsia (AOR = 1.75, 95% CI: 1.04, 2.95) (Table 2).

**Table 2:** Severity characteristics of the preeclampsia by onset of disease, Ayder comprehensive specialised hospital, Mekelle, Northern Ethiopia, 2017–2021 (n = 934).

Characteristic	Total (n = 934)	Onset		AOR (95% CI)	P-value
		Early (n = 253)	Late (n = 681)		
Preeclampsia with severity features, n (%)	605 (64.8)	210 (83.0)	395 (58.0)	3.53 (2.46, 5.07)	**<0.001**
Eclampsia, n (%)	142 (15.2)	38 (15.0)	104 (15.3)	*	
Headache, n (%)	417 (44.6)	152 (60.1)	265 (38.9)	1.52 (1.01, 2.28)	**0.043**
Blurred vision, n (%)	264 (28.3)	102 (40.3)	162 (23.8)	1.30 (0.85, 2.00)	0.230
Blindness, n (%)	10 (1.1)	2 (0.8)	8 (1.2)	*	
Epigastric pain, n (%)	257 (27.5)	101 (39.9)	156 (22.9)	1.28 (0.83, 2.00)	0.258
Pain in the right upper quadrant n (%)	146 (15.6)	61 (24.1)	85 (12.5)	1.12 (0.67, 1.88)	0.653
Vomiting, n (%)	70 (7.5)	28 (11.1)	42 (6.2)	1.05 (0.56, 1.95)	0.886
Hand swelling, n (%)	56 (6.0)	21 (8.3)	35 (5.1)	0.46 (0.16, 1.28)	0.136
Facial swelling, n (%)	83 (8.9)	37 (14.6)	46 (6.7)	2.73 (1.17, 6.39)	**0.020**
Abdominal wall edema, (%)	62 (6.6)	20 (7.9)	42 (6.2)	*	
Nasal bleeding, (%)	21 (2.2)	10 (4.0)	11 (1.6)	1.17 (0.40, 3.44)	0.772
Vulvar edema, (%)	40 (4.3)	16 (6.3)	24 (3.5)	1.06 (0.46, 2.46)	0.892
SBP above 160 mmHg, n (%)	498 (53.3)	156 (61.7)	342 (50.2)	1.07 (0.64, 1.79)	0.797
DBP above 110 mmHg, n (%)	412 (44.1)	139 (54.9)	273 (40.1)	1.71 (1.03, 2.84)	**0.037**
Creatinine greater than 1.3, n (%) (n = 829)	119 (14.3)	38 (16.2)	81 (13.6)	*	
Platelet less than 100,000, n (%) (n = 879)	174 (19.8)	55 (22.4)	119 (18.8)	*	
Hemoglobin level less than 11, n (%) (n = 898)	155 (17.3)	47 (18.9)	108 (16.6)	*	
ALT greater than 64, n (%) (n = 744)	138 (18.5)	50 (23.4)	88 (16.6)	0.91 (0.51, 1.61)	0.753
AST greater than 62, n (%) (n = 827)	197 (23.8)	72 (30.5)	125 (21.1)	1.75 (1.04, 2.95)	**0.035**

SBP: systolic blood pressure, DBP: diastolic blood pressure, AOR: adjusted odds ratio, AST: aspartate aminotransferase, ALT: alanine aminotransferase.

* Variables that did not meet the inclusion criteria for final multivariable model.

### Neonatal and maternal complications

Women with early-onset preeclampsia tend to have a longer duration of stay in the hospital compared to those with late onset (AOR = 4.70, 95% CI: 2.15, 10.28). Babies born to women with EO-PE were also more likely to have a lower APGAR score at the 5^th^ minute compared to their counterparts (AOR = 13.79, 95% CI: 1.16, 163.78). Similarly, unlike women with late onset preeclampsia, bad obstetric outcomes such as low birth weight (AOR = 10.14, 95% CI 4.29, 23.91) and neonatal mortality (AOR = 6.82, 95% CI: 1.89, 24.58) were observed among women with early onset (Table 3).

**Table 3:** Maternal and neonatal complications among women with early versus late onset preeclampsia., Ayder comprehensive specialised hospital, Mekelle, Northern Ethiopia, 2017–2021 (n = 934).

Characteristic	Total (n = 934)	Onset		AOR (95% CI)	P-value
		Early (n = 253)	Late (n = 681)		
Maternal complication, n (%)					
Renal insufficiency	123 (13.2)	38 (15.0)	85 (12.5)	*	
Liver involvement	223 (23.9)	82 (32.4)	141 (20.7)	1.25 (0.46, 3.41)	0.657
Neurologic complication	488 (52.2)	177 (70.0)	311 (45.7)	1.57 (0.74, 3.33)	0.241
Hematologic complication ^a^	182 (19.5)	58 (22.9)	124 (19.5)	1.02 (0.38, 2.72)	0.969
Thrombocytopenia	174 (19.8)	55 (22.4)	119 (18.8)	*	
DIC	10 (1.1)	4 (1.6)	6 (0.9)	*	
Hemolysis	100 (10.7)	34 (13.4)	66 (9.7)	0.47 (0.09, 2.31)	0.351
Oligohydramnios	80 (8.6)	30 (11.9)	50 (7.3)	0.94 (0.23, 3.87)	0.929
Abruption	58 (6.2)	17 (6.7)	41 (6.0)	*	
PPH	50 (5.3)	11 (4.3)	39 (5.7)	0.40 (0.06, 2.47)	0.324
Aspiration pneumonia	71 (7.6)	22 (8.7)	49 (7.2)	*	
Blood transfusion	70 (7.5)	20 (7.9)	50 (7.3)	*	
Length of stay greater the mean (7.7 days), n (%)	240 (25.7)	116 (45.8)	124 (18.2)	4.70 (2.15, 10.28)	**<0.001**
ICU admission, n (%)	40 (4.3)	14 (5.5)	26 (3.8)	*	
Maternal death, n (%)	25 (2.7)	6 (2.4)	19 (2.8)	*	
Mode of delivery, n (%)					
Spontaneous vaginal delivery	501 (53.6)	136 (53.7)	365 (53.6)	*	
Operative vaginal delivery	26 (2.8)	4 (1.6)	22 (3.2)	**	
Cesarean section	403 (43.1)	109 (43.1)	294 (43.2)	*	
Pregnancy outcomes, n (%)					
Stillbirth	122 (13.1)	68 (26.9)	54 (7.9)	1.38 (0.03, 55.48)	0.863
IUGR	113 (12.1)	43 (17.0)	70 (10.3)	0.98 (0.36, 2.71)	0.979
Neonatal complications, n (%)					
1^st^ minute APGAR less than 7	166 (19.1)	83 (37.9)	83 (12.8)	0.55 (0.13, 2.37)	0.424
5^th^ minute APGAR less than 7	105 (12.0)	62 (27.4)	43 (6.6)	13.79 (1.16, 163.78)	**0.038**
Birth weight less than 2.5 kg	429 (47.0)	215 (88.1)	214 (32.0)	10.14 (4.29, 23.91)	**<0.001**
Neonatal death	28 (6.0)	14 (12.8)	14 (3.9)	6.82 (1.89, 24.58)	**<0.001**

SD: standard deviation, SBP: systolic blood pressure, DBP: diastolic blood pressure, AOR: adjusted odds ratio, HELLP: elevated liver enzymes and low platelets, PPH: postpartum haemorrhage, ICU, intensive care unit, IUGR: intrauterine growth restriction, APGAR.

* Variables that didn’t fulfill the inclusion criteria for final multivariable model,

** Omitted because of collinearity,

^a^ Controlled for all but hemolysis and PPH

### Treatment of preeclampsia-eclampsia syndrome

In the present study, hydralazine was the most commonly used antihypertensive to manage both groups while magnesium sulfate was the most commonly used for prophylaxis and management among pregnant women with preeclampsia-eclampsia syndrome. Among 57 women with previous history of PE-E syndrome, less than half (n = 27, 47.4%) were on aspirin for prophylaxis to delay or prevent PE-E syndrome. Only slightly above half (n = 139, 54.9%) of the mothers in the early onset group received steroid for fetal lung maturity (Table 4).

**Table 4:** Management of preeclampsia and eclampsia, Ayder comprehensive specialized hospital, Mekelle, Northern Ethiopia, 2017–2021 (n = 934).

Medicine	Total (n = 934)	Onset	
		Early (n = 253)	Late (n = 681)
Antihypertensive			
Nifedipine	201 (21.5)	69 (27.3)	132 (19.4)
Methyldopa	184 (19.70)	83 (32.8)	101 (14.8)
Hydralazine	526 (56.3)	169 (66.8)	357 (52.4)
Lasix (furosemide)	50 (5.3)	21 (8.3)	29 (4.3)
Anticonvulsant use			
MgSO4	895 (95.8)	243 (96.0)	652 (95.7)
Diazepam	24 (2.6)	15 (5.9)	9 (1.3)
Asprin prophylaxis for the prevention of PE			
Aspirin	23 (2.5)	4 (1.6)	19 (2.8)
Steroid for lung maturity			
Dexamethasone	251 (26.9)	139 (54.9)	112 (16.4)

## Discussion

In this 7-year study, 27,350 mothers gave birth at Ayder Hospital, among them 1095 mothers had PE-E syndrome, with a prevalence of [4.0 (95% CI: 3.8, 4.2)]. The deaths of 25 mothers associated with PE-E syndrome were recorded. There was a statistically significant difference in terms of disease severity; severe disease was more common among the EO-PE group than in the LO-PE group. Adverse neonatal complications and neonatal death rates were also statistically higher in the EO-PE group than in the LO-PE group.

The prevalence of PE-E syndrome was higher than in studies conducted in the US (3.1%), Sweeden (2.9%), and China (2.3%) [18, 19]. However, it was similar to the prevalence of PE-E syndrome described in a systematic review in Ethiopia (4.74% (95% CI (3.99, 5.49)) [20]. Furthermore, the prevalence was the same as the WHO secondary analysis of PE-E syndrome in low- and middle income countries [21]. The slightly higher prevalence of PE-E syndrome in the present study as well as in other LMICs compared to the prevalence in high-income countries might be due to several factors. First, black race is a presumptive risk factor that increases the risk of developing PE-E syndrome, although current understanding favors structural racism as a risk factor instead of black race itself [22]. Second, micronutrient deficiencies that are widely prevalent in LMIC are thought to play a role in the PE-E pathogenesis [23]. Third, although not well elucidated, anemia, which is common in developing countries, is an incriminated factor that accelerates the progress of gestational hypertension to PE-E syndrome [24] giving rise to a high prevalence of preeclampsia.

Pre-eclampsia-eclampsia syndrome forms an infamous triad with obstetric hemorrhage and sepsis and greatly contributes to maternal, fetal, and neonatal mortality, particularly in resource constrained settings [8, 25, 26]. In general, 25 (2.7%) mothers with preeclampsia-eclampsia syndrome died in the present study. A systematic review and meta-analysis of maternal and neonatal outcomes of hypertensive disorders in Ethiopia found a similar pooled maternal death prevalence of 4% (95% CI: 2, 6%) [27]. This record of high maternal mortality is similar to findings of other studies conducted in low-resource settings both in Africa and elsewhere [28, 29]. The prevalence of maternal death among women complicated by preeclampsia recorded in this study is higher than the findings in middle and high-income settings [30–32]. The higher prevalence of maternal mortality associated with PE-E syndrome in low resource settings could be related to late presentation of patients associated with lack of proper knowledge, limited access to quality antenatal care [33, 34], and poor obstetric care provision in health institutions due to resource constraints in such settings [35]. Moreover, in the study setting availability of intensive care is limited [26] and the only available intensive care unit is reported to have a high level of mortality [36]. Moreover, The shortage of blood and the blood products and lack of appropriate antibiotics to treat simultaneously occurring and/or complicating PE-E syndrome are prevalent in the study setting. These might have contributed to the staggering prevalence of maternal mortality in the study setting. The two groups did not have statistically significant differences in terms of maternal mortality, highlighting that neither form of PE should be considered benign. Previous Interventions in developing countries have shown that maternal mortality is preventable wit equitable implementation of quality improvement initiatives to recognize and promptly treat hypertensive disorders and to increase awareness of early warning signs of the disease [37].

Risk factors such as extreme age (≤18 or ≥ 35 years) at conception, having a history of medical disorders namely; chronic hyperension, diabetes melites (both gestational and overt), thyroid disorders and chronic kidney disease were more prevalent in the EO-PE group than in the LO-PE group. On the other had, primigravidity, having mulerian anomaly, obesity at conception, and prior history of preeclampsia-eclampsia syndrome were more attributable to the LO-PE group than the EO-PE. However, statistically speaking, the two groups did not differ in terms of attributable risk factors. This was similar to another study conducted elsewhere [38]. In contrast, different studies have demonstrated that EO-PE and LO-PE could be different in respect to attributable risk factors [12, 39, 40]. Although different in terms of the prevalence of attributable risk factors between the two groups in the present work, the failure to attain statistical significance might be related to the nature of the data used in this study. Since this is a retrospective review of medical records where the collection of patient history is not structured, little attention may have been paid to dig out risk factors that are remotely relevant to the case management.

Preeclampsia-eclampsia syndrome is also associated with myriads of severe maternal complications. The present study revealed that women with EO-PE tend to develop significant severity features. Severity features such as headache, facial edema, abdominal wall edema, and diastolic blood pressure 110 showed significant association with EO-PE (p-value <0.005). In extreme cases the disease can lead to kidney, liver failure, DIC, and central nervous system disorders [41]. Similarly, in the present study neurologic, liver, hematologic, and renal dysfunctions were recorded in 52.2%, 23.9%, 19.5% and 13.2% of women respectively. The extent of these serious maternal complications warrants the need for a multidisciplinary approach that includes senior obstetricians, intensivists, hematologists and nephrologists in the management of such high-risk mothers. Although it does not reach the statical significance, women in the EO-PE group had consistently higher rates of organ-system dysfunction than the women in the LO-PE group. These findings were in agreement with other studies elsewhere [12, 42].

Previous studies have demonstrated that hypertensive disorders are attended with a high burden of adverse perinatal outcomes in Ethiopia [19, 43–46]. The results of the present study supported this observation. The overall rates of stillbirth and neonatal mortality in this study were 13.1% and 6.0%, respectively. Almost half (47.0%) of neonates had a birth weight <2.5 K.g., 12.1% had IUGR, and the APGAR score was <7 in 12.0% of the neonates. The frequency of adverse perinatal outcomes is generally high in the sub-Saharan African setting [12, 47] compared to resource-rich setting [18]. The high burden of perinatal morbidity and mortality in low-resource setting like ours can be attributed to the high rate of indicated preterm deliveries in these high-risk women in a setting that lacks both personnel and technologies to handle delicate preterm babies. The high frequency of indicated preterm birth associated with preeclampsia results in preterm babies with deficient surfactant that make it more difficult for the lung to ventilate [19]. When such deliveries are anticipated, all the necessary equipment and well-trained personnel for newborn resuscitation should be readily available. In low-resource settings, such readiness is infrequently fulfilled. For example, the use of surfactant therapy was not available in the study setting during the study period. Recent evidence shows that administration of magnesium sulphate for neuroprotection in the context of imminent preterm birth at <31^+6^ weeks could favor a relatively better neonatal outcome [48]. This practice as well is lacking in the study area. The higher burden of unfavorable perinatal outcome in women with PE-E syndrome in the present study could be due to such deficiencies in the standard of care.

Previous studies show that overall adverse perinatal outcomes are higher in women with EO-PE than in women with LO-PE [39]. Similarly, in the present study, the low fifth minute APGAR score (27.4% vs 6.6%), birth weight less than 2.5 kg (88.1% Vs 32.0%), and the rate of neonatal death (12.8% Vs 3.9%) were significantly higher in the EO-PE group than in the LO-PE group (p-value <0.05). This was in congruence with previous studies which similarly reported significantly higher rates of poor APGAR, low birth weight, and neonatal death rates in EO-PE compared to LO-PE [38, 49]. Several similar studies also reported higher neonatal morbidity and mortality in EO-PE than LO-PE [4, 39, 50–52]. Significant perinatal adverse outcomes associated with EO-PE can be due to the associated high frequency of SGA alone or another unsolved pathophysiologic mechanism that warrants further investigation [53].

## Limitations

Out study has several limitations. First, substantial sociodemographic variables were missing from the patient charts. Demographic variables such as educational status, marital status, level of education, and occupation were not routinely recorded in patient charts. Second, women who are classified as LO-PE might have their onset earlier than 34 weeks but are classified as LO-PE because the mothers present late. Third, neonatal status was not routinely recorded in patient notes during postpartum follow up. The neonatal mortality recorded in this study mainly reflects the death of neonates until the mother is discharged from the hospital. In the discharge letter, both the neonatal and maternal status is routinely recorded. It should also be noted some inherent caveats of retrospective cohort such as; missing information when using existing records (information bias) or by selection bias, because individuals are selected after the outcome has occurred, so the presence of both conditions (exposure and outcome) present at the moment of data collection might have affected our study Finally, reporting OR in a cohort study is a limitation, as it shows the association not the causation.

## Conclusion

The present study showcases the difference in between EO-PE and LO-PE in terms of clinical presentation, maternal and perinatal unfavorable outcomes. Women with EO-PE had significantly higher odds of developing severe clinical manifestations and end organ dysfunction than their LO-PE counter parts. In general, perinatal morbidity and mortality was also increased significantly in EO-PE. Therefore, gestational age at the time of the disease should be taken as an important indicator of disease severity that leads to poor maternal and perinatal outcomes. The maternal mortality recorded in this study is staggering. Statistically speaking, both groups contributed to maternal death equally, revealing that both types of diseases should not be considered benign. To this end, since both diseases are not benign, careful clinical vigilance is required in all women presenting with PE-E syndrome, especially with EO-PE where a critical decision-making is required to balance the risk of delivery (to avoid adverse outcome due to a progressive disease) versus expectant management (to avoid prematurity). Overall, EO-PE is related to significantly higher maternal and neonatal adverse outcomes. According to contemporary recommendations to prevent or delay the onset of preeclampsia [54], initiation of prophylactic administration of low-dose daily low dose aspirin (81–150 mg) between 12 weeks and 28 weeks of gestation, optimally before 16 weeks (11–14 weeks), and continued until delivery might improve maternal and neonatal outcomes of women at risk of developing EO-PE [55–58].

## Supporting information

S1 Checklist STROBE statement—Checklist of items that should be included in reports of observational studies. (DOCX)

S1 Data (DTA)
